# Antimicrobial Resistance in Selected Foodborne Pathogens in Sub-Saharan Africa: A Systematic Review and Meta-Analysis

**DOI:** 10.3390/antibiotics15010087

**Published:** 2026-01-15

**Authors:** Kedir A. Hassen, Jose Fafetine, Laurinda Augusto, Inacio Mandomando, Marcelino Garrine, Gudeta W. Sileshi

**Affiliations:** 1 Department of Animal and Public Health, Faculty of Veterinary (FAVET), Eduardo Mondlane University (UEM), Maputo 1102, Mozambique; jose.fafetine@uem.mz (J.F.); laurinda.augusto@uem.mz (L.A.); 2 Centre of Excellence in Agri-Food Systems and Nutrition (CE-AFSN), Eduardo Mondlane University (UEM), Praça 25 de Junho C.Posta 257 Edificio da Reitoria 5° Andar, Maputo 1102, Mozambique; sileshigw@gmail.com; 3 Centro de Investigação em Saúde de Manhiça (CISM), Maputo 1929, Mozambique; inacio.mandomando@manhica.net (I.M.); marcelino.garrine@manhica.net (M.G.); 4 Global Health and Tropical Medicine—GHTM, Associate Laboratory in Translation and Innovation Towards Global Health—LA-REAL, Instituto de Higiene e Medicina Tropical—IHMT, Universidade NOVA de Lisboa—UNL, 2829-516 Lisbon, Portugal; 5 Department of Plant Biology and Biodiversity Management, College of Natural and Computational Sciences, Addis Ababa University (AAU), Addis Ababa P.O. Box 3434, Ethiopia

**Keywords:** evidence synthesis, antimicrobial resistance, zoonosis, one health, knowledge–policy integration, sub-Saharan Africa

## Abstract

**Background/Objectives:** The increasing trend of foodborne zoonotic pathogens exhibiting antimicrobial resistance (AMR) represents a growing threat to food safety and public health in sub-Saharan Africa (SSA). Resistant strains of foodborne zoonotic pathogens compromise treatment efficacy, raise illness, and threaten sustainable food systems in human and animal health. However, regional understanding and policy response are limited due to the fragmentation of data and the inadequacy of surveillance. This systematic review and meta-analysis aimed to achieve the following: (1) estimate the pooled prevalence of AMR, including multidrug resistance (MDR) in selected foodborne pathogens; (2) compare subgroup variations across countries, pathogen species, and antibiotic classes; and (3) evaluate temporal trends. **Methods:** Following PRISMA 2020 guidelines, studies published between 2010 and June 2025 reporting AMR and MDR in *Salmonella*, *Campylobacter*, or *E. coli* from food or animal sources in SSA were systematically reviewed. Data on pathogen prevalence, AMR profile, and MDR were extracted. Random-effects meta-analysis using R software was implemented to estimate the pooled prevalence and the 95% confidence intervals (95% CI). Subgroup analyses were performed to explore heterogeneity across countries, antibiotic class, and bacterial species. **Results:** Ninety studies from 16 sub-Saharan African countries were included, encompassing 104,086 positive isolates. The pooled foodborne pathogen prevalence was 53.1% (95% CI: 51.5–54.7), AMR prevalence was 61.6% (95% CI: 59.4–63.9), and MDR prevalence was 9.1% (95% CI: 8.3–10.0). The highest resistance was reported in *Campylobacter* spp. (43.6%), followed by *Salmonella* spp. (29.1%) and *E. coli* (22.8%). High heterogeneity was observed across studies (*I*^2^ = 95–99%, *p* < 0.001). **Conclusions:** It is concluded that substantial AMR burden exists in food systems, highlighting an urgent need for integrated One Health surveillance, antimicrobial stewardship, and policy harmonization in SSA. Strengthening laboratory capacity, enforcing prudent antimicrobial use, and promoting regional data sharing are critical for the management of antimicrobial resistance in sub-Saharan Africa.

## 1. Introduction

Sub-Saharan Africa (SSA) faces a dual threat to food security and public health due to the growing burden of antimicrobial resistance (AMR) and foodborne zoonoses [1,2,3,4]. Increasing resistance has been documented in critical pathogens such as *Escherichia coli*, *Klebsiella pneumoniae*, and *Staphylococcus aureus* [5,6]. Likewise, zoonotic bacteria such as *Salmonella* spp., *Campylobacter* spp., and *Brucella* spp. continue to cause widespread disease, contributing to high morbidity, mortality, and economic losses in both livestock and human populations [7,8,9,10].

Antimicrobial resistance in enteric bacteria originating from livestock and food products represents an escalating global challenge that threatens the effectiveness of antimicrobial therapy, food safety, and trade [11,12,13,14]. In sub-Saharan Africa, livestock production plays a vital role in supporting nutrition, food security and rural incomes, making the impact of antimicrobial resistance negatively significant [15,16,17]. The emergence and spread of resistant bacteria are driven by unregulated antimicrobial use, poor farm biosecurity, limited veterinary supervision, and weak surveillance and laboratory facilities [18,19,20,21].

The misuse of antimicrobials in animal production, commonly for prophylaxis, treatment, and growth promotion, exerts selective pressure that promotes resistance [22,23,24,25]. Because many veterinary drugs share similar chemical structures or mechanisms of action with those used in human medicine, cross-resistance further accelerates this process [26,27,28,29]. Additional drivers include the circulation of counterfeit human medicines, informal veterinary drug markets, and limited diagnostic capacity across sub-Saharan Africa [30,31,32,33].

Globally, AMR has been recognized as a major public health emergency with far-reaching health, social, and economic consequences [34]. The dwindling development pipeline for new antibiotics underscores the urgency of preserving existing drug efficacy [35,36]. Livestock such as cattle, small ruminants, poultry, and swine can serve as reservoirs of resistant bacteria that may spread to humans through direct contact, consumption of contaminated animal products, or exposure to contaminated environments [37,38]. Addressing this challenge requires an integrated One Health framework that links human, animal, and environmental health systems, recognizing that zoonotic bacteria can persist in animal waste, can contaminate soil and water, and subsequently infect humans through food or environmental exposure [39,40].

International organizations such as the Food and Agriculture Organization (FAO), the World Organization for Animal Health (WOAH), and the World Health Organization (WHO) have called for a coordinated global action plan on antimicrobial resistance, and prudent use of antimicrobials in humans, foods, and animal health sectors [41]. These emphasize policy coherence, harmonized surveillance, and cross-sectoral collaboration, given that an estimated 60% of emerging infectious diseases in humans originate from animals [42,43].

Despite global and regional initiatives, the actual prevalence and drivers of AMR in food systems remain poorly characterized in SSA due to fragmented data, limited surveillance, and research gaps [44,45]. This lack of comprehensive evidence hinders the development of context-specific interventions and policy responses [46,47,48,49]. Understanding the distribution, patterns, and determinants of resistance in key foodborne pathogens is therefore critical to designing effective One Health strategies [50,51,52,53].

This systematic review and meta-analysis synthesize current evidence on the prevalence and distribution of antimicrobial resistance in selected foodborne pathogens isolated from food animals and food products in sub-Saharan Africa. Specifically, the study aims to: (1) estimate the pooled prevalence of AMR, including multidrug resistance (MDR) in selected foodborne pathogens; (2) compare subgroup variations across countries, pathogen species, and antibiotic classes; and (3) evaluate temporal trends (2010–June 2025). Collectively, the findings from these analyses are expected to provide an evidence base to guide regional policy and interventions aimed at mitigating the emergence and spread of AMR, including MDR in SSA food systems.

## 2. Results

### 2.1. Characteristics of Included Studies and Their Distributions

**Figure 1:**
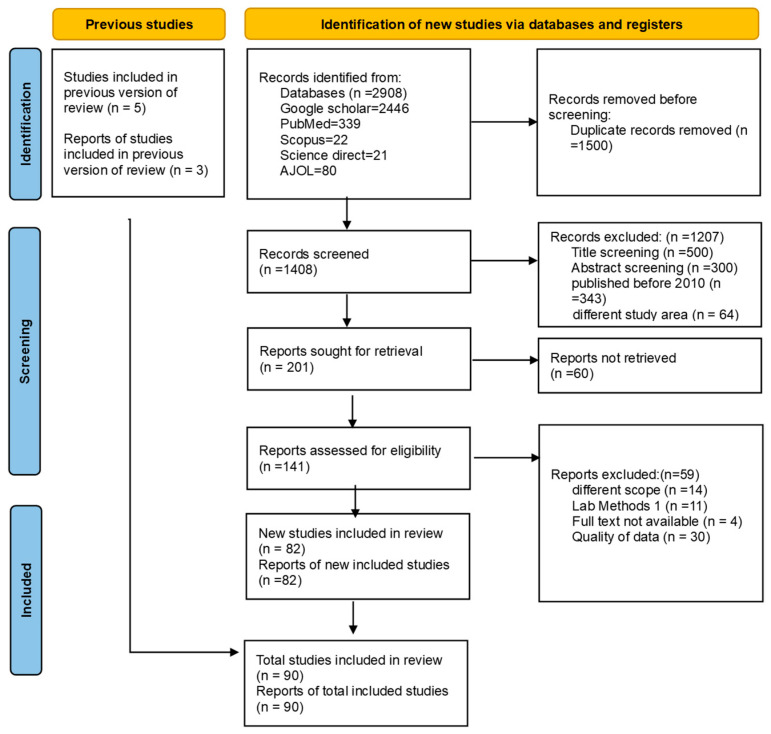
PRISMA flow chart showing the identification, screening, and included studies.

A total of 90 published articles encompassing 1555 observations were included in the quantitative synthesis. Figure 1 shows the number of studies identified and selected for inclusion in the review and meta-analysis. The PRISMA checklist guidelines were provided in the Supplementary Material S1. The PRISMA 2020 flow diagram reporting the selection process across databases, registers, and other sources is shown in Figure 1 [54].

**Figure 2:**
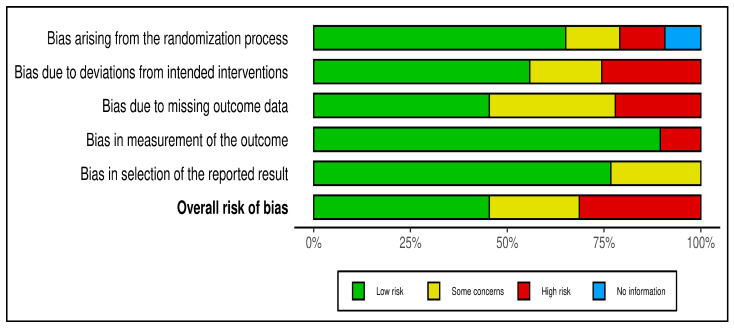
Summary of risk of bias assessment.

To evaluate methodological quality, the Cochrane risk of bias assessment tool (RoB 2.0) was used. The full report of risk of bias across studies, with detailed study-level assessments, was presented in Supplementary Figure S1. The summary of risk of bias across studies was reported as shown in Figure 2 below.

**Figure 3:**
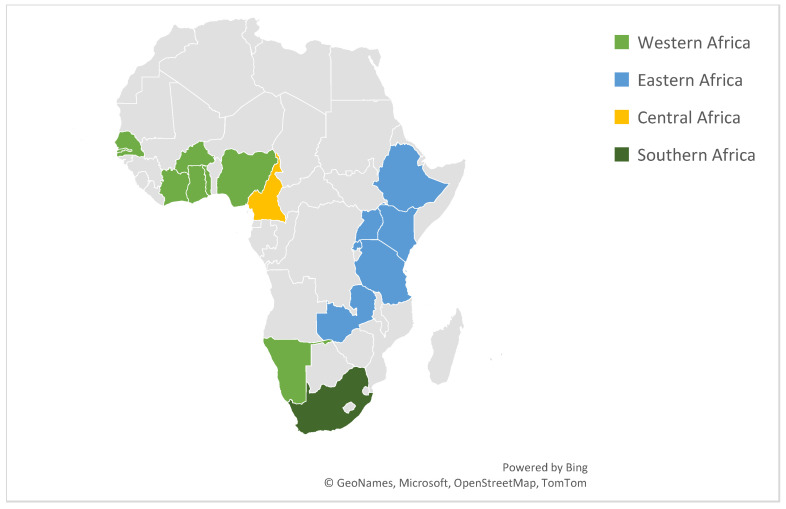
Map of sub-Saharan Africa showing the distribution of regions.

In the regional analysis, Eastern Africa contributed the largest number (n) of studies (n = 50/90, 55.5%), followed by Western Africa (n = 28/90; 31.1%), Southern Africa (n = 11/90; 12.2%), and Central Africa (n = 1/90; 1.1%). This irregular distribution indicates a research imbalance across the continent, as shown in the sub-Saharan Africa Maps in Figure 3, from (2010–June 2025).

Out of the 90 studies, 41, 31, and 30 studies have reported *Salmonella* spp., Pathogenic *E. coli*, and *Campylobacter* spp. (Table 1). The dataset comprising 104,086 positive isolates. Pathogenic *E. coli* accounted for the largest share (n = 58,231), followed by *Campylobacter* spp. (n = 27,073) and *Salmonella* spp. (n = 18,782). The summary of included studies by pathogen is shown in Table 1.

### 2.2. Pathogen Prevalence

The pooled random-effects model estimated that 53.1% (95% CI: 51.5–54.7) of food animal and product samples were contaminated with pathogenic *E. coli*, *Salmonella* spp., or *Campylobacter* spp., with substantial heterogeneity across studies (*I*^2^ = 99.0%, *τ*^2^ = 0.1030, *p* < 0.001). Contamination levels varied significantly by pathogen (*Q* = 262.4, *p* < 0.0001). Pathogenic *E. coli* had a pooled prevalence of 44.0%, compared with 52.0% for non-pathogenic *E. coli*. *Campylobacter* spp. and *Salmonella* spp. showed lower prevalence estimates of 18.4% and 17.6%, respectively. Evidence of publication bias is shown in Supplementary Figure S2a.

#### 2.2.1. Subgroup Analysis of Pathogen Prevalence

According to the subgroup analysis, pathogen prevalence varied significantly across countries (*Q* = 616.7, *p* < 0.0001). The highest prevalence was recorded in Uganda (85.6%) followed by Tanzania (66.9%), Senegal (37.7%) and Togo (34.3%), whereas the lowest prevalence was found in Gambia (2.6%) and Ghana (9.5%). The subgroup analysis of pathogen prevalence across SSA countries is shown in Table 2.

#### 2.2.2. Temporal Trend of Pathogen Prevalence

The meta-regression analysis also revealed a significant positive temporal trend in pathogen prevalence across the 1555 observations (*β* = 0.0765, SE = 0.0098, *z* = 7.77, *p* < 0.0001), but with high residual heterogeneity (*I*^2^ = 98.9%, *τ*^2^ = 2.8867). The reported prevalences have increased substantially over time, as shown in Table 3.

### 2.3. Antimicrobial Resistance (AMR) Prevalence

The pooled prevalence of antimicrobial Resistance was 61.6% (95% CI: 59.4–63.9), indicating that nearly two-thirds of bacterial isolates were resistant to at least one antimicrobial. Heterogeneity remained substantial (*I*^2^ = 99.5%, *τ*^2^ = 0.1950, *p* < 0.001). Supplementary Figure S2b shows funnel plot asymmetry indicating publication bias.

#### 2.3.1. Subgroup Analysis of AMR Prevalence Rate by Country

In the subgroup analysis, AMR prevalence followed a similar pattern, with Cameroon (63.1%), Ghana (55.3%), Nigeria (50.5%), Uganda (45.9%), and Tanzania (41.5%) reporting the highest resistance levels, while Namibia (4.2%) showed the lowest. The subgroup analysis of AMR prevalence across SSA countries was reported as shown in Table 4.

#### 2.3.2. Subgroup Analysis of AMR Prevalence by Pathogen Type

Regarding AMR, *Campylobacter* spp. displayed the highest resistance (43.6%, 95% CI: 40.2–46.9%), followed by *Salmonella* spp. (29.1%) and *E. coli* (22.8%), suggesting *Campylobacter* as a major resistance reservoir in food systems. The subgroup analysis of AMR prevalence by pathogen type is shown in Table 5.

#### 2.3.3. Subgroup Analysis of AMR Prevalence Rate by Classes

In the subgroup analysis, resistance patterns varied markedly among antimicrobial classes (*Q* = 44,512.2, *p* < 0.0001). Resistance levels varied widely. Rifamycins showed the highest resistance (100%), followed by polypeptide antibiotics (88%) and glycopeptides (73%). High resistance was also observed in tetracyclines (54%) and folate pathway inhibitors (50%). Moderate resistance levels were found for Nitrofurans (40%), Macrolides (39%), and Lincosamides (36%). Lower resistance proportions were noted for β-Lactam antibiotics (35%), Fluoroquinolones (24%), Aminoglycosides (23%), Phenicols (20%), and Polymyxins (9%). The subgroup analysis of AMR prevalence by antimicrobial classes is shown in Table 6.

#### 2.3.4. Temporal Trend of AMR Prevalence by Year

The mixed-effects meta-regression of 1552 observation AMR estimates showed very high heterogeneity (*τ*^2^ = 4.62; *I*^2^ = 98.68%). Year significantly influenced resistance levels (*Q* = 36.74, *p* < 0.0001), revealing a clear upward trend. AMR increased by approximately 0.08 units per year (estimate = 0.0785, *p* < 0.0001), indicating a steady rise in resistance among foodborne pathogens over time. The reported AMR prevalences have increased substantially over time, as shown in Table 7.

### 2.4. Multidrug Resistance (MDR) Prevalence

The overall pooled prevalence of MDR was 9.1% (95% CI: 8.3–10.0), with high heterogeneity (*I*^2^ = 95.5%, *τ*^2^ = 0.0262, *p* < 0.001). A total of 1253 isolates from 16 African countries were included in the analysis. The prevalence of multidrug resistance (MDR) varied widely across countries. Cameroon reported the highest MDR prevalence (53.3%), followed by Rwanda (7.2%), Uganda (4.3%), Tanzania (4.1%), Nigeria (3.6%), Ethiopia (3.3%), Zambia (1.8%), South Africa (1.2%), and Kenya (0.3%). In contrast, no MDR isolates were reported in Burkina Faso, Côte d’Ivoire, Gambia, Ghana, Namibia, Senegal, or Togo. These findings highlight substantial heterogeneity in MDR occurrence across the region.

A total of 1553 isolates from three major foodborne pathogens were included in the analysis. *Escherichia coli* showed the highest multidrug-resistance (MDR) prevalence at 4.2%, followed by *Salmonella* spp. at 2.3%. *Campylobacter* spp. had the lowest MDR prevalence at 1.7%. Overall, the data indicate relatively low but variable levels of MDR across the different pathogens. Supplementary Figure S2c shows the corresponding funnel plot for MDR estimates.

## 3. Discussion

### 3.1. Pathogen Prevalence

The results show that over half of food animals and their product samples were contaminated with zoonotic bacteria, underscoring the persistence of hygiene and biosecurity gaps along the food chain. The higher prevalence in sub-Saharan Africa is probably a reflection of weak enforcement of Good Agricultural Practices (GAPs) and Good Manufacturing Practices (GMPs), limited regulatory supervision, inadequate training for producers, and insufficient investment in hygiene and biosecurity infrastructure across the livestock value chain [55,56,57,58]. The dominance of pathogenic *E. coli*, *Campylobacter* spp., and *Salmonella* spp., all major causes of human gastroenteritis, reflects their endemic circulation in livestock and their food products [59,60,61]. Poor slaughter hygiene, informal meat handling, and limited cold-chain capacity likely drive these trends [62,63,64]. The observed high heterogeneity (*I*^2^ > 98%) suggests substantial methodological and ecological variability across countries, emphasizing the urgent need for standardized surveillance and laboratory protocols across sub-Saharan Africa [65,66,67].

Significant geographic variation was observed across the region. Eastern Africa contributed the largest dataset and exhibited the highest contamination rates. Countries such as Tanzania and Uganda showed markedly higher pathogen prevalence, likely due to informal slaughter practices and limited veterinary oversight. Country-level variations in AMR prevalence likely reflect disparities in veterinary infrastructure, antimicrobial regulation, laboratory capacity, and surveillance systems. Nations with limited veterinary supervision, weak policy enforcement, and inadequate diagnostic resources tend to report higher resistance rates. Due to a lack of sufficient data, this review could not sufficiently explore the relative importance of these drivers in determining pathogen prevalence. In contrast, Namibia and The Gambia recorded lower contamination rates, probably reflecting stronger regulatory enforcement and veterinary infrastructure. The substantial between-country heterogeneity reflects uneven implementation of antimicrobial stewardship, disparities in farm-level biosecurity, and gaps in national surveillance frameworks [68,69].

The meta-regression revealed a significant upward trend in pathogen prevalence published between 2010 and June 2025, suggesting a worsening contamination burden over time. This trajectory parallels Africa’s rapid livestock intensification, urbanization, and expansion of informal food markets. The persistently high residual heterogeneity further implies that unmeasured contextual factors, such as geography and methodology, antimicrobial access, biosecurity enforcement, and laboratory diagnostic capacity, contribute to contributed to variability. These findings highlight the importance of longitudinal and genomic surveillance to track resistance emergence and transmission dynamics more accurately. The upward trend underscores a growing burden of foodborne pathogens and antimicrobial resistance across sub-Saharan Africa, emphasizing the urgent need for integrated surveillance and policy action [70].

### 3.2. Antimicrobial Resistance (AMR) Prevalence

The pooled AMR prevalence of 61.6% demonstrates extensive bacterial exposure to antimicrobials throughout the food system. High resistance observed against polypeptides, glycopeptides, penicillin, first-generation cephalosporins, aminopenicillins, tetracyclines, and folate pathway inhibitors indicates excessive and unregulated antibiotic use in veterinary production. This misuse is driven by easy access without prescription, lack of farmer awareness on antimicrobial stewardship, limited veterinary services, and economic pressures to enhance growth and prevent disease under poor biosecurity conditions [71]. The higher AMR proportion observed in *Campylobacter* spp. (43.6%) compared with *Salmonella* (29.1%) and *E. coli* (22.8%) should be interpreted cautiously, as differences between species may reflect variable study designs, sampling methods, and selective pressures rather than true biological differences. These findings are consistent with reports from Ethiopia, Kenya, and Tanzania showing similar resistance trends in poultry and cattle isolates [72,73,74,75,76,77]. The pervasive resistance within foodborne bacteria not only reduces livestock productivity but also threatens the efficacy of essential human antibiotics [78,79,80,81]. These findings reinforce WHO and Africa CDC assessments recognizing Africa as a global AMR hotspot [82,83,84,85].

This review shows high levels of antimicrobial resistance across several major drug classes, with the greatest resistance observed in Rifamycins, polypeptide antibiotics, and glycopeptides. Resistance to commonly used agents such as tetracyclines and folate pathway inhibitors further emphasizes the growing challenge of effective treatment and demonstrates the extensive misuse of broad-spectrum antimicrobials in livestock production. The substantial heterogeneity among studies suggests large variations in resistance patterns across regions and study settings. Strengthened surveillance and improved antimicrobial stewardship are urgently needed to address these trends. Due to the very small sample size, the high AMR prevalence found for glycopeptides, polypeptides, and Rifamycins needs to be interpreted cautiously, while low resistance to carbapenems, phosphonic acids, and polymyxins suggests these agents remain relatively effective. Emerging resistance patterns raise concern for the future of last-resort antibiotics. Similar trends have been reported across East Africa, where carbapenem-producing Enterobacteriaceae have begun to emerge in both clinical and agricultural contexts [86,87,88,89]. The nearly complete resistance to rifamycin observed in limited datasets signals a potential new frontier in resistance evolution that warrants urgent genomic investigation. This finding reflects widespread misuse of antimicrobials in livestock production systems and weak stewardship enforcement, constituting a major One Health threat [90,91,92].

### 3.3. Multi Drug Resistance (MDR) Prevalence

The pooled prevalence of multidrug resistance, although lower than single-drug resistance, remains concerning due to the association of MDR with mobile genetic elements that facilitate horizontal gene transfer across bacterial species and environments [93,94,95,96]. Comparable meta-analyses from sub-Saharan Africa report MDR prevalence between 8 and 15%, confirming that resistant pathogens are widespread across clinical, animal, and food sources [97,98,99,100]. The high heterogeneity likely reflects differences in study design, bacterial species, antimicrobial testing, and antibiotic use practices across countries [101,102,103]. Even a modest MDR burden poses significant risks by reducing treatment options, prolonging illness, and increasing healthcare costs. Strengthening antimicrobial stewardship, diagnostic capacity, and genomic surveillance under the One Health framework is critical to track and contain MDR dissemination.

This review highlights notable differences in multidrug-resistance (MDR) patterns among major foodborne pathogens. *Escherichia coli* exhibited the highest MDR prevalence (4.2%), reflecting its frequent exposure to diverse antimicrobial agents in both human and animal settings, which may accelerate resistance development. *Salmonella* spp. showed moderate MDR levels (2.3%), consistent with documented regional variation in antimicrobial use practices along the food production chain. *Campylobacter* spp. demonstrated the lowest MDR prevalence (1.7%), although the organism’s intrinsic resistance mechanisms and challenges in laboratory recovery may contribute to underestimation.

Overall, although the MDR prevalence appears relatively low, the observed differences across pathogens suggest varying ecological pressures, antimicrobial usage behaviors, and surveillance sensitivity. These findings underscore the continued need for harmonized pathogen-specific monitoring and targeted interventions across the One Health spectrum to prevent further emergence and spread of resistance.

### 3.4. Policy Implications

The high prevalence of antimicrobial resistance (AMR) and the presence of multidrug resistance highlight an urgent need for coordinated One Health policy actions that integrate human, animal, and environmental health sectors. MDR strains, frequently driven by mobile genetic elements such as plasmids, transposons, and integrons, pose a serious challenge to treatment efficacy and food safety, underscoring the need for strengthened surveillance and antimicrobial stewardship across all levels of the food chain. Regional alignment with the WHO Global Antimicrobial Resistance Surveillance System (GLASS) and the Africa CDC AMR Surveillance Network (AMRSNET) is crucial for data harmonization, quality assurance, and evidence-based policy development [104]. Key policy priorities should include: Strengthening national legislation regulating veterinary antimicrobial sales and use; Enforcing sanitary and biosecurity standards across meat, dairy, and poultry value chains; Expanding diagnostic, genomic, and metagenomic surveillance infrastructure to monitor MDR and resistance gene dissemination; and Implementing risk communication and community education programs targeting farmers, butchers, and food vendors. Such integrated and evidence-driven interventions are vital to contain AMR and MDR dissemination, safeguard food security, and preserve regional trade integrity.

### 3.5. Limitations and Future Directions

This study was constrained by the reliance on secondary data with heterogeneous methodologies and potential publication bias. The definition and reporting of multidrug resistance (MDR) varied considerably across studies, which limited comparability and prevented a meaningful subgroup or trend analysis. Nevertheless, the inclusion of a large pooled dataset and application of robust random-effects models enhances the credibility and generalizability of the findings. In the past, some food additives containing heavy metals have also been linked with AMR, but we could not establish this in this systematic review due to the scarcity of data. Future research should employ whole-genome sequencing (WGS) and metagenomic approaches to comprehensively characterize AMR determinants, MDR plasmid transmission pathways, and virulence factors. The limited number of studies from Central Africa restricts regional representativeness and may bias pooled estimates. This highlights the need for increased investment in AMR surveillance and coordinated research networks across underrepresented regions to inform equitable policy decisions [44]. Strengthening laboratory capacity, data integration, and policy translation within the One Health framework will be key to achieving sustainable AMR and MDR control in sub-Saharan Africa [45].

## 4. Materials and Methods

### 4.1. Study Design and Protocol Registration

A systematic review and meta-analysis were conducted to estimate the pooled prevalence of antimicrobial resistance (AMR), multidrug resistance (MDR), and foodborne bacterial pathogens (*Salmonella* spp., pathogenic *E. coli*, and *Campylobacter* spp.) in food-producing animals and food products across sub-Saharan Africa (SSA). The review adhered to the Preferred Reporting Items for Systematic Reviews and Meta-Analyses (PRISMA) guidelines [54] and was registered with PROSPERO (ID: 1151530). This study was structured following the PICOTS framework:

- Population (P): Farm animals (cattle, poultry, pigs, goats, and sheep) and their derived food products (meat, milk, and eggs).
- Intervention/Exposure (I): Exposure to antimicrobial-resistant enteric pathogens (*Salmonella* spp., pathogenic *E. coli*, *Campylobacter* spp.).
- Comparator (C): Not applicable to prevalence analysis.
- Outcomes (O): Pooled prevalence of AMR, MDR, and pathogen isolation rates; resistance profiles by antibiotic class.
- Time (T): Studies published between 2010 and June 2025.
- Setting (S): Farms, slaughterhouses, markets, and retail outlets across SSA.

The geographical scope followed the United Nations [105], macro-regional classification, dividing SSA into Eastern, Central, Southern, and Western Africa.

### 4.2. Search Strategy

A comprehensive literature search was conducted in PubMed, Scopus, ScienceDirect, Google Scholar, and African Journals Online (AJOL) for studies published between 2010 and June 2025. Boolean operators and Medical Subject Headings (MeSH) were applied as follows: (“antimicrobial Resistance” OR “antimicrobial Resistance” OR “multidrug resistance”) AND (“enteric pathogens” OR “*Salmonella*” OR “*Escherichia coli*” OR “*Campylobacter*”) AND (“meat” OR “milk” OR “eggs”) AND (“livestock” OR “farm animals” OR “poultry” OR “cattle” OR “pigs” OR “goats”) AND (“Sub-Saharan Africa” OR “East Africa” OR “West Africa” OR “Southern Africa” OR “Central Africa”).

Reference lists of eligible studies were screened to identify additional publications. When full-text access was unavailable, corresponding authors were contacted. Only English-language, peer-reviewed studies meeting the inclusion criteria were retained. Two reviewers independently screened titles, abstracts, and full texts, resolving disagreements through discussion.

### 4.3. Eligibility Criteria

Studies were included if they reported primary data on antimicrobial resistance and MDR in *Salmonella* spp., *Escherichia coli*, or *Campylobacter* spp. from food-producing animals or food products in SSA. Non-indexed sources, such as institutional reports and local journals, were screened through Google Scholar and relevant repositories, provided they contained sufficient methodological detail and laboratory-based results. Reviews, editorials, and duplicate datasets were excluded.

The inclusion criteria were as follows:

1. Reported laboratory-confirmed isolates of *Salmonella* spp., pathogenic *E. coli*, or *Campylobacter* spp.
2. Originated from food-producing animals or derived food products (meat, milk, eggs, carcasses, ready-to-eat meat, cheese, or sausages).
3. Employed standardized antimicrobial susceptibility testing (AST) methods (disk diffusion, broth/agar dilution, E-test, MIC, or VITEK) with interpretation based on CLSI or EUCAST guidelines.
4. Provided quantitative data on AMR and/or MDR prevalence.

The exclusion criteria are as follows:

- Focused on non-bacterial pathogens (parasites, fungi, or viruses).
- Included wildlife, companion animals, or aquatic species.
- Were reviews, editorials, conference abstracts, or gray literature.
- Lacked explicit AMR/MDR data or prevalence estimates.
- Not published in English.

### 4.4. Data Extraction

Data were extracted using a standardized Excel form, including first author, publication year, country, study design, animal species, sample type (meat, milk, feces, swab), bacterial species, AST methods, antibiotics tested, and interpretation guidelines.

Quantitative variables extracted were as follows:

- Sample size and number of positive isolates;
- Pathogen prevalence;
- AMR and MDR rates (MDR defined as resistance to ≥3 antibiotic classes);
- Geographic region and study setting.

All duplicate records retrieved from Google Scholar and other databases were removed using Mendeley software, Excel, and manual screening before title and abstract review. Reference management was performed using Mendeley (version X7; Thomson Reuters, Toronto, ON, Canada).

### 4.5. Quality Assessment

Study quality was evaluated using the Cochrane Risk of Bias Tool (RoB 2.0), adapted for observational studies. Three domains were assessed as follows:

- Selection bias: representativeness of study population;
- Measurement bias: reliability of laboratory testing and AST interpretation;
- Reporting bias: completeness and transparency of data.

Each study was rated as having low, moderate, or high risk of bias. Discrepancies between reviewers were resolved by consensus.

### 4.6. Data Synthesis and Statistical Analysis

Random effects meta-analysis of proportions was performed using the meta and metafor packages of the R software (version 4.3.1). Pooled prevalence estimates and their 95% confidence intervals (CIs) were computed using DerSimonian–Laird random-effects models to account for inter-study variability. For assessing heterogeneity between studies or categories, Cochran’s *Q*, the heterogeneity variance (*τ*^2^), and *I*^2^ statistics were used. These were generated by the random effects models. Cochran’s *Q* provides a test of the hypothesis that the true treatment effects are the same in all the primary studies included in a meta-analysis. Normally, *τ*^2^ is used as a measure of the amount of true variance in the effect sizes across the population of studies. When the value of *I*^2^ is 0, all variability in effect size estimates is assumed to be due to sampling error within studies, while *I*^2^ > 50% indicates significant heterogeneity between studies. Following the same logic, we interpreted *I*^2^ > 50% as an indication of significant heterogeneity between studies or subgroups. In addition, subgroup analyses were performed to manage heterogeneity. The subgroups were based on (1) bacterial pathogen (*Salmonella* spp., pathogenic *E. coli*, *Campylobacter* spp.); (2) antibiotic classes (such as β-lactams, fluoroquinolones, tetracyclines, and others); and (3) country and subregion (Eastern, Western, Southern, Central Africa).

Publication bias was assessed using Egger’s regression test, the Trim-and-Fill method, and visual funnel plots. Pooled AMR and MDR event rates were presented with 95% CIs, weighted by the inverse of study variance. For ease of interpretation, all proportions and their 95% CIs were converted from proportions to percentage values. All statistical scripts, datasets, and analytical codes are available upon request or through the corresponding author.

### 4.7. Ethical Considerations and AI Disclosure

This study analyzed data from previously published literature; therefore, ethical approval was not required. The review adhered to open science principles for data sharing and transparency. Generative Artificial Intelligence (GenAI), specifically, ChatGPT (OpenAI GPT-5), was used for language editing, formatting standardization, and structure alignment with the MDPI Antibiotics guidelines. No AI was used to generate, modify, or analyze the data.

## 5. Conclusions

It is concluded that a high burden of antimicrobial resistance and multidrug resistance exists among *Salmonella* spp., *Escherichia coli*, and *Campylobacter* spp. from food-producing animals and food products in sub-Saharan Africa. The high AMR prevalence and MDR rate highlight a serious One Health challenge with significant implications for food safety, public health, and regional trade. It is also concluded that resistance is most pronounced against certain antibiotics. Rifamycins showed the highest resistance, followed by polypeptide antibiotics and glycopeptides, tetracyclines, and folate pathway inhibitors, underscoring the need for prudent antimicrobial use and stronger surveillance systems. Addressing these threats requires harmonized AMR monitoring, effective regulation of veterinary antimicrobial use, and investment in diagnostic and genomic capacity to detect and control resistance early. Sustained One Health collaboration and evidence-driven policies are essential to preserve antimicrobial efficacy, protect public health, and ensure sustainable agri-food systems across sub-Saharan Africa.

## Figures and Tables

**Table 1:** Summary of included studies by pathogen species.

Pathogen	No. Studies (n)	N	Positive Isolates	Pathogen (%)
*Salmonella* spp.	41	96,721	18,782	17.6
Pathogenic *E. coli*	31	119,859	58,231	44.0
*Campylobacter* spp.	30	175,704	27,073	18.4
Total	90	392,284	104,086	53.1

**Table 2:** Subgroup analysis of pathogen prevalence results by country.

			Pathogen Prevalence	Heterogeneity		
Country	Regions	k ^†^	in % (95% CI)	*τ* ^2^	*I*^2^ (%)	*Q*
Burkina Faso	West Africa	68	21.0 (17.6–24.6)	0.0205	87.4	532.6
Cameroon	Central Africa	12	22.4 (14.2–31.8)	0.0256	92.8	152.14
Cote d’Ivoire	West Africa	23	33.1 (25.0–41.6)	0.0402	97.0	732.05
Ethiopia	East Africa	296	24.7 (21.1–28.4)	0.1317	99.4	52,828.1
Gambia	West Africa	12	2.6 (2.6–2.6)	0.0	0.0	0.0
Ghana	West Africa	156	9.5 (8.1–11.0)	0.0223	97.3	5703.03
Kenya	East Africa	172	21.0 (17.8–24.4)	0.0695	98.6	12,029.7
Namibia	Southern Africa	7	32.9 (29.9–36.0)	0.0002	12.7	6.87
Nigeria	West Africa	201	21.8 (18.8–25.0)	0.064	96.2	5215.42
Rwanda	East Africa	77	21.3 (19.1–23.7)	0.0145	95.0	1515.27
Senegal	West Africa	11	37.7 (37.7–37.7)	0.0	0.0	0.0
South Africa	Southern Africa	231	20.1 (17.6–22.8)	0.0571	97.4	8764.12
Tanzania	East Africa	122	66.9 (61.4–72.2)	0.0972	97.8	5384.47
Togo	West Africa	88	34.3 (27.8–41.0)	0.1019	97.8	3903.23
Uganda	East Africa	38	85.6 (62.3–97.4)	0.2877	99.3	2884.89
Zambia	Southern Africa	41	23.7 (0.141–0.348)	0.1461	99.4	6891.92

^†^ k represents the number of observations.

**Table 3:** Temporal trend of pathogen prevalence by year.

Parameter	Estimate (*β*)	SE	*z*-Value	*p*-Value	95% CI
Intercept	−155.75	19.88	−7.84	<0.0001	−194.71 to −116.80
Year	0.0765	0.0098	7.77	<0.0001	0.0572–0.0958

**Table 4:** Subgroup analysis of AMR prevalence rate by country.

			AMR Prevalence	Heterogeneity		
Country	Region	k ^†^	in % (95% CI)	*τ* ^2^	*I*^2^ (%)	*Q*
Burkina Faso	West Africa	68	26.4 (19.0–34.5)	0.1205	98.7	5129.95
Cameroon	Central Africa	12	63.1 (27.1–92.4)	0.3334	99.4	1852.05
Côte d’Ivoire	West Africa	23	25.3 (15.0–37.1)	0.0849	98.2	1219.93
Ethiopia	East Africa	296	30.9 (26.3–35.6)	0.1865	99.3	43,293.93
Gambia	West Africa	12	38.4 (11.9–69.3)	0.2454	99.8	5415.81
Ghana	West Africa	156	55.3 (47.8–62.7)	0.2264	99.8	62,372.45
Kenya	East Africa	172	28.4 (23.6–33.4)	0.1272	99.1	18,972.39
Namibia	Southern Africa	7	4.2 (0.1–12.9)	0.0278	96.3	164.30
Nigeria	West Africa	201	50.5 (44.3–56.8)	0.1972	99.3	29,634.48
Rwanda	East Africa	77	14.5 (8.6–21.7)	0.1675	99.6	20,743.17
Senegal	West Africa	11	18.7 (6.4–35.6)	0.0799	98.9	893.40
South Africa	Southern Africa	231	25.6 (20.5–31.1)	0.2128	99.5	45,963.07
Tanzania	East Africa	122	41.5 (34.9–48.4)	0.1419	99.3	16,562.13
Togo	West Africa	88	22.0 (15.1–29.8)	0.1698	98.4	5300.42
Uganda	East Africa	54	45.9 (28.2–65.4)	0.1557	98.5	1783.2
Zambia	Southern Africa	41	25.6 (14.2–39.0)	0.2025	99.6	9920.30

^†^ k represents the number of observations.

**Table 5:** Subgroup analysis of AMR prevalence by pathogen type.

		AMR Prevalence	Heterogeneity		
Bacteria	k ^†^	in % (95% CI)	*τ* ^2^	*I*^2^ (%)	*Q*
*Campylobacter* spp.	660	43.5 (40.2–46.9)	0.1929	99.6	150,877.5
*Escherichia coli*	417	22.8 (19.6–26.2)	0.1631	99.4	70,412.52
*Salmonella* spp.	478	29.1 (25.5–32.9)	0.1977	99.3	71,610.12

^†^ k represents the number of observations.

**Table 6:** Subgroup Analysis AMR prevalence rate by Classes.

		AMR Prevalence			
Antimicrobial Class	k ^†^	In % (95% CI)	*τ* ^2^	*I*^2^ (%)	*Q*
Aminoglycosides	257	23.1 (19.4–27.0)	0.1287	99.1	27,097.3
Fluoroquinolones	280	23.7 (19.8–27.8)	0.1526	99.3	38,331.27
Folate Pathway Inhibitors	136	49.8 (42.6–56.9)	0.1758	99.5	25,708.75
Glycopeptides	6	72.6 (29.9–98.9)	0.1746	99.6	1157.82
Lincosamides	4	35.6 (20.2–52.7)	0.0114	96.1	76.24
Macrolides	100	39.4 (30.3–48.8)	0.2254	99.7	30,606.17
Nitrofurans	18	40.5 (19.8–63.0)	0.2056	99.6	3995.84
Phenicol’s	96	20.0 (14.2–26.4)	0.1355	99.4	14,651.03
Phosphonic Acids	2	5.3 (0.00–100.0)	0.0323	95.7	23.51
Pleuromutilins	1	39.8 (33.9–45.9)	0.0	0.0	0.0
Polymyxins	8	9.0 (0.00–40.5)	0.194	98.1	368.98
Polypeptide Antibiotics	2	88.0 (0.06–100.0)	0.0148	88.9	9.02
Rifamycin’s	2	100.0 (98.7–100.0)	0.0	0.0	0.02
Tetracyclines	169	53.5 (46.8–60.2)	0.1894	99.5	32,620.34
β-Lactam Antibiotics	471	34.7 (30.6–38.8)	0.2247	99.6	119,206.85

^†^ k represents the number of observations.

**Table 7:** Temporal trend of AMR prevalence by year.

Parameter	Estimate (*β*)	SE	*z*-Value	*p*-Value	95% CI
Intercept	−161.6759	26.1326	−6.1868	<0.0001	−212.8948 to −110.4570
Year	0.0785	0.0129	6.0617	<0.0001	0.0531 to 0.1038

## Data Availability

All data supporting the findings of this study are available within the article and its Supplementary Materials. Extracted data and analytical code (R scripts) are available from the corresponding author upon the reviewer’s and editor’s request.

## Supplementary Materials

The following supporting information can be downloaded at: https://www.mdpi.com/article/10.3390/antibiotics15010087/s1, S1. PRISMA checklist showing study selection process; Figure S1. Risk of bias assessment full result. Figure S2: Funnel plots results assessing publication bias.

## Abbreviations

The following abbreviations were used in this manuscript:

Abbreviation	Definition
AMR	Antimicrobial Resistance
MDR	Multidrug Resistance
AST	Antimicrobial Susceptibility Testing
CLSI	Clinical and Laboratory Standards Institute
EUCAST	European Committee on Antimicrobial Susceptibility Testing
SSA	Sub-Saharan Africa
*E. coli*	*Escherichia coli*
WHO	World Health Organization
FAO	Food and Agriculture Organization of the United Nations
WOAH	World Organization for Animal Health (formerly OIE)
GLASS	Global Antimicrobial Resistance Surveillance System
AMRSNET	Africa Antimicrobial Resistance Surveillance Network
LMICs	Low- and Middle-Income Countries
PICOTS	Population, Intervention, Comparator, Outcome, Timeframe, and Setting
PRISMA	Preferred Reporting Items for Systematic Reviews and Meta-Analyses
PROSPERO	International Prospective Register of Systematic Reviews
*Q*	Cochran’s Heterogeneity Statistic
*I*^2^	I-squared (Measure of Statistical Heterogeneity)
CI	Confidence Interval
Β	Regression Coefficient
One Health	Integrated Approach Linking Human, Animal, and Environmental Health

## References

[1] Kalule J.B., Smith A., Vulindhlu M., Tau N., Nicol M., Keddy K., Robberts L. (2019). Prevalence and antibiotic susceptibility patterns of enteric bacterial pathogens in human and non-human sources in an urban informal settlement in Cape Town, South Africa. BMC Microbiol..

[2] Matakone M., Founou R.C., Founou L.L., Dimani B.D., Koudoum P.L., Fonkoua M.C., Boum-Ii Y., Gonsu H., Noubom M. (2024). Multi-drug resistant (MDR) and extended-spectrum β-lactamase (ESBL) producing *Escherichia coli* isolated from slaughtered pigs and slaughterhouse workers in Yaoundé, Cameroon. One Health.

[3] Okumu N.O., Muloi D.M., Moodley A., Ochien’G L., Watson J., Kiarie A., Ngeranwa J.J., Cumming O., Cook E.A. (2025). Epidemiology of Antimicrobial-Resistant Diarrheagenic *Escherichia coli* Pathotypes from Children, Livestock and Food in Dagoretti South, Nairobi Kenya. Int. J. Antimicrob. Agents.

[4] Ronald C., Matofari J.W., Nduko J.M. (2023). Antimicrobial resistance of *E. coli* strains in ready-to-eat red meat products in Nakuru County, Kenya. Microbe.

[5] Zeru F., Adamu H., Woldearegay Y.H., Tessema T.S., Hansson I., Boqvist S. (2025). Occurrence, risk factors and antimicrobial resistance of *Campylobacter* from poultry and humans in central Ethiopia: A one health approach. PLoS Negl. Trop. Dis..

[6] Njoga E.O., Mshelbwala P.P., Ogugua A.J., Enemuo-Edo E.C., Onwumere-Idolor O.S., Ogunniran T.M., Bernard S.N., Ugwunwarua J.C., Anidobe E.C., Okoli C.E. (2025). *Campylobacter* Colonisation of Poultry Slaughtered at Nigerian Slaughterhouses: Prevalence, Antimicrobial Resistance, and Risk of Zoonotic Transmission. Trop. Med. Infect. Dis..

[7] Beyene A.M., Alemie Y., Gizachew M., E Yousef A., Dessalegn B., Bitew A.B., Alemu A., Gobena W., Christian K., Gelaw B. (2024). Serovars, virulence factors, and antimicrobial resistance profile of non-typhoidal *Salmonella* in the human-dairy interface in Northwest Ethiopia: A one health approach. PLoS Negl. Trop. Dis..

[8] Dieye Y., Hull D.M., Wane A.A., Harden L., Fall C., Sambe-Ba B., Seck A., Fedorka-Cray P.J., Thakur S. (2022). Genomics of human and chicken *Salmonella* isolates in Senegal: Broilers as a source of antimicrobial resistance and potentially invasive nontyphoidal salmonellosis infections. PLoS ONE.

[9] Mokgophi T.M., Gcebe N., Fasina F., Adesiyun A.A. (2021). Antimicrobial resistance profiles of *Salmonella* isolates on chickens processed and retailed at outlets of the informal market in Gauteng Province, South Africa. Pathogens.

[10] Okello P., Bjöersdorff O.G., Hansson I., Boqvist S., Erume J. (2025). Prevalence and antimicrobial resistant *Campylobacter* spp. in broiler chicken carcasses and hygiene practises in informal urban markets in a low-income setting. PLoS ONE.

[11] Kagambèga A.B., Dembélé R., Traoré O., Wane A.A., Mohamed A.H., Coulibaly H., Fall C., Bientz L., M’Zali F., Mayonnove L. (2024). Isolation and Characterization of Environmental Extended Spectrum β-Lactamase-Producing *Escherichia coli* and *Klebsiella pneumoniae* from Ouagadougou, Burkina Faso. Pharmaceuticals.

[12] French N.P., Thomas K.M., Amani N.B., Benschop J., Bigogo G.M., Cleaveland S., Fayaz A., Hugho E.A., Karimuribo E.D., Kasagama E. (2024). Population Structure and Antimicrobial Resistance in *Campylobacter jejuni* and *C. coli* Isolated from Humans with Diarrhea and from Poultry, East Africa. Emerg. Infect. Dis..

[13] Dekker D., Eibach D., Boahen K.G., Akenten C.W., Pfeifer Y., Zautner A.E., Mertens E., Krumkamp R., Jaeger A., Flieger A. (2019). Fluoroquinolone-Resistant *Salmonella enterica*, *Campylobacter* spp., and *Arcobacter butzleri* from Local and Imported Poultry Meat in Kumasi, Ghana. Foodborne Pathog. Dis..

[14] Mainda G., Bessell P.R., Muma J.B., McAteer S.P., Chase-Topping M.E., Gibbons J., Stevens M.P., Gally D.L., Bronsvoort B.M.d.C. (2015). Prevalence and patterns of antimicrobial resistance among *Escherichia coli* isolated from Zambian dairy cattle across different production systems. Sci. Rep..

[15] Karikari A.B., Obiri-Danso K., Frimpong E.H., Krogfelt K.A. (2017). Antibiotic Resistance of *Campylobacter* Recovered from Faeces and Carcasses of Healthy Livestock. Biomed. Res. Int..

[16] Kambuyi K. (2018). Occurrence and Antimicrobial Resistance of *Campylobacter* spp. Isolates from Beef Cattle in Gauteng and North West Provinces, South Africa. Master’s Thesis.

[17] Kagambèga A., Thibodeau A., Trinetta V., Soro D.K., Sama F.N., Bako É., Bouda C.S., N’diaye A.W., Fravalo P., Barro N. (2018). *Salmonella* spp. and *Campylobacter* spp. in poultry feces and carcasses in Ouagadougou, Burkina Faso. Food Sci. Nutr..

[18] Akinduti A.P., Ayodele O., Motayo B.O., Obafemi Y.D., Isibor P.O., Aboderin O.W. (2022). Cluster analysis and geospatial mapping of antibiotic resistant *Escherichia coli* O157 in southwest Nigerian communities. One Health.

[19] Muligisa-Muonga E., Mainda G., Mukuma M., Kwenda G., Hang’ombe B., Flavien B.N., Phiri N., Mwansa M., Munyeme M., Muma J.B. (2021). Antimicrobial resistance of *Escherichia coli* and *Salmonella* isolated from retail broiler chicken carcasses in Zambia. J. Epidemiol. Res..

[20] Olabode H.O., Mailafia S., Ogbole M.E., Okoh G.R., Ifeanyi C.I., Onigbanjo H.O., Ugbaja I.B. (2017). Isolation and Antibiotic Susceptibility of *Campylobacter* Species from Cattle Offals in Gwagwalada Abattoir, Abuja-FCT Nigeria. Int. J. Curr. Microbiol. Appl. Sci..

[21] Ogbor O., Ajayi A., Zautner A.E., Smith S.I. (2019). Antibiotic susceptibility profiles of *Campylobacter coli* isolated from poultry farms in Lagos Nigeria—A pilot study. Eur. J. Microbiol. Immunol..

[22] Abubakar M.K., Muigai A.W.T., Ndung’u P., Kariuki S. (2019). Investigating Carriage, Contamination, Antimicrobial Resistance and Assessment of Colonization Risk Factors of *Campylobacter* spp. in Broilers from Selected Farms in Thika, Kenya. Microbiol. Res. J. Int..

[23] Gahamanyi N., Habimana A.M., Harerimana J.P., Mukayisenga J., Ntwali S., Umuhoza A., Nsengiyumva E., Irimaso E., Shimirwa J.B., Pan C.-H. (2025). Antimicrobial susceptibility profiles of thermophilic *Campylobacter* species from human, pig, and chicken feces in Rwanda. Front. Microbiol..

[24] Wesonga S.M., Muluvi G.M., Okemo P.O., Kariuki S. (2010). Antibiotic resistant *Salmonella* and *Escherichia coli* isolated from indiginous *Gallus domesticus* in Nairobi, Kenya. East Afr. Med. J..

[25] Uaboi-Egbenni P.O., Bessong P.O., Samie A., Obi C.L. (2012). Potentially pathogenic *Campylobacter* species among farm animals in rural areas of Limpopo province, South Africa: A case study of chickens and cattles. Afr. J. Microbiol. Res..

[26] Uaboi-Egbenni P.O., Bessong P.O., Samie A., Obi C.L. (2011). Prevalence, haemolysis and antibiograms of *Campylobacters* isolated from pigs from three farm settlements in Venda region, Limpopo province, South Africa. Afr. J. Biotechnol..

[27] Uaboi-Egbenni P.O., Bessong P.O., Samie A., Obi C.L. (2011). Prevalence and antimicrobial susceptibility profiles of *Campylobacter jejuni* and coli isolated from diarrheic and non-diarrheic goat faeces in Venda region, South Africa. Afr. J. Biotechnol..

[28] Uaboi-Egbenni P.O., Bessong P.O., Samie A., Obi C.L. (2010). Campylobacteriosis in sheep in farm settlements in the Vhembe district of South Africa. Afr. J. Microbiol. Res..

[29] Salihu M.D., Junaidu A.U., Magaji A.A., Yakubu Y. (2012). Prevalence and Antimicrobial Resistance of Thermophilic *Campylobacter* Isolates from Commercial Broiler Flocks in Sokoto, Nigeria. Res. J. Vet. Sci..

[30] Rene K.A., Adjehi D., Timothee O., Tago K., Marcelin D.K., Ignace-Herve M.E. (2014). Serotypes and antibiotic resistance of *Salmonella* spp. isolated from poultry carcass and raw gizzard sold in markets and catering in Abidjan, Côte d’Ivoire. Int. J. Curr. Microbiol. Appl. Sci.

[31] Okunlade A.O., Ogunleye A.O., Jeminlehin F.O., Ajuwape A.T.P. (2015). Occurrence of *Campylobacter* species in beef cattle and local chickens and their antibiotic profiling in Ibadan, Oyo State, Nigeria. Afr. J. Microbiol. Res..

[32] Nonga H.E., Muhairwa A.P. (2010). Prevalence and antibiotic susceptibility of thermophilic *Campylobacter* isolates from free range domestic duck (*Cairina moschata*) in Morogoro municipality, Tanzania. Trop. Anim. Health Prod..

[33] Nigatu S., Mequanent A., Tesfaye R., Garedew L. (2015). Prevalence and Drug Sensitivity Pattern of *Campylobacter jejuni* Isolated from Cattle and Poultry in and Around Gondar Town, Ethiopia. Glob. Vet..

[34] Resource Type Themes Keywords African Union AMR Landmark Report: Voicing African Priorities on the Active Pandemic. https://africacdc.org/download/african-union-amr-landmark-report-voic.

[35] Food and Agriculture Organization of the United Nations (2021). The FAO Action Plan on Antimicrobial Resistance 2021–2025.

[36] Velazquez-Meza M.E., Galarde-López M., Carrillo-Quiróz B., Alpuche-Aranda C.M. (2022). Antimicrobial resistance: One Health approach. Vet. World.

[37] World Organisation for Animal Health (2020). OIE Standards, Guidelines and Resolution on Antimicrobial Resistance and the Use of Antimicrobial Agents.

[38] Sartorius B., Gray A.P., Weaver N.D., Aguilar G.R., Swetschinski L.R., Ikuta K.S., Mestrovic T., Chung E., Wool E.E., Han C. (2024). The burden of bacterial antimicrobial resistance in the WHO African region in 2019: A cross-country systematic analysis. Lancet Glob. Health.

[39] FAO, OIE, WHO (2019). Monitoring and Evaluation of the Global Action Plan on Antimicrobial Resistance Framework and Recommended Indicators.

[40] Essack P.S., Essack S.Y. (2025). AMR Surveillance in Africa: Are We There Yet?. Int. J. Infect. Dis..

[41] Kajumbula H.M., Amoako D.G., Tessema S.K., Aworh M.K., Chikuse F., Okeke I.N., Okomo U., Jallow S., Egyir B., Kanzi A.M. (2024). Enhancing clinical microbiology for genomic surveillance of antimicrobial resistance implementation in Africa. Antimicrob. Resist. Infect. Control.

[42] Onohuean H., Olot H., Onohuean F.E., Bukke S.P.N., Akinsuyi O.S., Kade A. (2025). A scoping review of the prevalence of antimicrobial-resistant pathogens and signatures in ready-to-eat street foods in Africa: Implications for public health. Front. Microbiol..

[43] Wada N., Todo A. Civil Society Report on Sustainable Development Goals: Agenda 2030. Sustainable Development Goals: Agenda 2030 A Civil Society Report INDIA 2017. https://www.gcap.global/wp-content/uploads/2018/07/Sustainable-Development-Goals-2018.pdf.

[44] Antimicrobial Resistance Control—Africa CDC. https://africacdc.org/antimicrobial-resistance/.

[45] Strategic Framework for Collaboration on Antimicrobial Resistance. https://www.who.int/publications/i/item/9789240045408.

[46] Mathole M.A., Muchadeyi F.C., Mdladla K., Malatji D.P., Dzomba E.F., Madoroba E. (2017). Presence, distribution, serotypes and antimicrobial resistance profiles of *Salmonella* among pigs, chickens and goats in South Africa. Food Control.

[47] Komba E.V.G., Mdegela R.H., Msoffe P.L.M., Matowo D.E., Maro M.J. (2014). Occurrence, species distribution and antimicrobial resistance of thermophilic *Campylobacter* isolates from farm and laboratory animals in Morogoro, Tanzania. Vet. World.

[48] Iroha I.R., Ugbo E.C., Ilang D.C., Oji A.E., Ayogu T.E. (2011). Bacteria contamination of raw meat sold in Abakaliki, Ebonyi State Nigeria. J. Public Health Epidemiol..

[49] Garedew L., Hagos Z., Addis Z., Tesfaye R., Zegeye B. (2015). Prevalence and antimicrobial susceptibility patterns of *Salmonella* isolates in association with hygienic status from butcher shops in Gondar town, Ethiopia. Antimicrob. Resist. Infect. Control.

[50] Ewnetu D., Mihret A. (2010). Prevalence and Antimicrobial Resistance of *Campylobacter* Isolates from Humans and Chickens in Bahir Dar, Ethiopia. Foodborne Pathog. Dis..

[51] Chanyalew Y., Asrat D., Amavisit P., Loongyai W. (2013). Prevalence and Antimicrobial Susceptibility of Thermophilic *Campylobacter* Isolated from Sheep at Debre Birhan, North-Shoa, Ethiopia. Nat. Sci..

[52] Bester L.A., Essack S.Y. (2012). Observational study of the prevalence and antibiotic resistance of *Campylobacter* spp. from different poultry production systems in KwaZulu-Natal, South Africa. J. Food Prot..

[53] Bekele B., Ashenafi M. (2010). Distribution of drug resistance among enterococci and *Salmonella* from poultry and cattle in Ethiopia. Trop. Anim. Health Prod..

[54] Page M.J., McKenzie J.E., Bossuyt P.M., Boutron I., Hoffmann T.C., Mulrow C.D., Shamseer L., Tetzlaff J.M., Akl E.A., Brennan S.E. (2021). The PRISMA 2020 statement: An updated guideline for reporting systematic reviews. BMJ.

[55] Bawa I., Tchamba G.B., Bagre T., Bouda S., Konate A., Bako E., Kagambega A., Zongo C., Somda M., Savadogo A. (2015). Antimicrobial susceptibility of *Salmonella enterica* strains isolated from raw beef, mutton and intestines sold in Ouagadougou, Burkina Faso. J. Appl. Biosci..

[56] Bata S.I., Karshima N.S., Yohanna J., Dashe M., Pam V.A., Ogbu K.I. (2016). Isolation and antibiotic sensitivity patterns of *Salmonella* species from raw beef and quail eggs from farms and retail outlets in Jos, Plateau State, Nigeria. J. Vet. Med. Anim. Health.

[57] Alemu S., Zewde B.M. (2012). Prevalence and antimicrobial resistance profiles of *Salmonella enterica* serovars isolated from slaughtered cattle in Bahir Dar, Ethiopia. Trop. Anim. Health Prod..

[58] Ajayi A.O., Egbebi A.O. (2011). Antibiotic sucseptibility of *Salmonella* Typhi and *Klebsiella pneumoniae* from poultry and local birds in Ado-Ekiti, Ekiti-State, Nigeria. Ann. Biol. Res..

[59] Adeyanju G.T., Ishola O. (2014). *Salmonella* and *Escherichia coli* contamination of poultry meat from a processing plant and retail markets in Ibadan, Oyo state, Nigeria. Springerplus.

[60] Yemisi A.O., Oyebode A.T., Margaret A.A., Justina J.B. (2011). Prevalence of Arcobacter, *Escherichia coli*, Staphylococcus aureus and *Salmonella* species in Retail Raw Chicken, Pork, Beef and Goat meat in Osogbo, Nigeria. Sierra Leone J. Biomed. Res..

[61] Addis Z., Kebede N., Worku Z., Gezahegn H., Yirsaw A., Kassa T. (2011). Prevalence and antimicrobial resistance of *Salmonella* isolated from lactating cows and in contact humans in dairy farms of Addis Ababa: A cross sectional study. BMC Infect. Dis..

[62] Jibril A.H., Sarkinfulani S.A., Ballah F.M., Ibrahim A.M., Garba B., Garba S., Abubakar Y. (2025). Molecular prevalence and resistance profile of *Escherichia coli* from poultry in Niger State, Nigeria. J. Agric. Environ..

[63] Fashae K., Ogunsola F., Aarestrup F.M., Hendriksen R.S. (2010). Antimicrobial susceptibility and serovars of *Salmonella* from chickens and humans in Ibadan, Nigeria. J. Infect. Dev. Ctries..

[64] Baluka S.A., Musisi L.N., Buyinza L.S.Y., Ejobi F. (2021). Prevalence and Antimicrobial Resistance of Foodborne Pathogens in Sentinel Dairy Farms. Eur. J. Agric. Food Sci..

[65] FAO, OIE, WHO (2019). Monitoring Global Progress on Antimicrobial Resistance: Tripartite AMR Country Self-Assessment Survey.

[66] Collignon P.C., Conly J.M., Andremont A., McEwen S.A., Aidara-Kane A. (2016). World Health Organization Advisory Group on Integrated Surveillance of Antimicrobial Resistance (WHO-AGISAR). World Health Organization Ranking of Antimicrobials According to Their Importance in Human Medicine: A Critical Step for Developing Risk Management Strategies to Control Antimicrobial Resistance from Food Animal Production. Clin. Infect. Dis..

[67] Africa CDC (2024). Antimicrobial Resistance Surveillance Guidance for the African Region.

[68] WHO (2015). Global Action Plan on Antimicrobial Resistance.

[69] Founou L.L., Founou R.C., Essack S.Y. (2021). Antimicrobial Resistance in the farm-to-plate Continuum—More Than a Food Safety Issue. Future Sci. OA.

[70] WHO, FAO, OIE (2019). World Health Organization Collaboration with OIE on Foodborne Antimicrobial Resistance: Role of the Environment, Crops and Biocides.

[71] WHO (2017). Prioritization of Pathogens to Guide Discovery, Research and Development of New Antibiotics for Drug-Resistant Bacterial Infections, Including Tuberculosis.

[72] Nyabundi D., Onkoba N., Kimathi R., Nyachieo A., Juma G., Kinyanjui P., Kamau J. (2017). Molecular characterization and antibiotic resistance profiles of *Salmonella* isolated from fecal matter of domestic animals and animal products in Nairobi. Trop. Dis. Travel Med. Vaccines.

[73] Agumah N.B., Effendi M.H., Tyasningsih W., Witaningrum A.M., Ugbo E.N., Agumah O.B., Ahmad R.Z., Ugbo A.I., Nwagwu C.S., Khairullah A.R. (2025). Distribution of cephalosporin-resistant *Campylobacter* species isolated from meat sold in Abakaliki, Nigeria. Open Vet. J..

[74] Gemeda B.A., Wieland B., Alemayehu G., Knight-Jones T.J.D., Wodajo H.D., Tefera M., Kumbe A., Olani A., Abera S., Amenu K. (2023). Antimicrobial Resistance of *Escherichia coli* Isolates from Livestock and the Environment in Extensive Smallholder Livestock Production Systems in Ethiopia. Antibiotics.

[75] Katakweba A.A.S., Muhairwa A.P., Lupindu A.M., Damborg P., Rosenkrantz J.T., Minga U.M., Mtambo M.M.A., Olsen J.E. (2018). First Report on a Randomized Investigation of Antimicrobial Resistance in Fecal Indicator Bacteria from Livestock, Poultry, and Humans in Tanzania. Microb. Drug Resist..

[76] Touglo K., Djeri B., Sina H., Boya B., Ayadokoun G.G., Baba-Moussa L., Ameyapoh Y. (2025). First detection of resistance genes and virulence factors in *Escherichia coli* and *Salmonella* spp. in Togo: The case of imported chicken and frozen by-products. BMC Microbiol..

[77] Yashim B.J.S., Ssajjakambwe P., Ntulume I., Pius T. (2024). *Escherichia coli* isolated from raw cow milk at selected collection centres in Bushenyi district, Western Uganda: A potential risk to human health. Res. Sq..

[78] Manishimwe R., Moncada P.M., Musanayire V., Shyaka A., Scott H.M., Loneragan G.H. (2021). Antibiotic-resistant *Escherichia coli* and *Salmonella* from the feces of food animals in the east province of Rwanda. Animals.

[79] Nyolimati C.A., Mayito J., Obuya E., Acaye A.S., Isingoma E., Kibombo D., Byonanebye D.M., Walwema R., Musoke D., Orach C.G. (2025). Prevalence and factors associated with multidrug resistant *Escherichia coli* carriage on chicken farms in west Nile region in Uganda: A cross-sectional survey. PLoS Glob. Public Health.

[80] André P.B., Essodina T., Malibida D., Adeline D., Rianatou B.A. (2020). Antibiotic resistance of enterobacteria (*Escherichia coli*, *Klebsiella* spp. and *Salmonella* spp.) isolated from healthy poultry and pig farms in peri-urban area of Lome, Togo. Afr. J. Microbiol. Res..

[81] Maina J., Ndung’u P., Muigai A., Kiiru J. (2021). Antimicrobial resistance profiles and genetic basis of resistance among non-fastidious Gram-negative bacteria recovered from ready-to-eat foods in Kibera informal housing in Nairobi, Kenya. Access Microbiol..

[82] Sime M.G. (2021). Occurrence and Antimicrobial Susceptibility of *Salmonella* in Fecal and Carcass Swab Samples of Small Ruminants at Addis Ababa Livestock Market. J. Vet. Sci..

[83] Eguale T., Birungi J., Asrat D., Njahira M.N., Njuguna J., Gebreyes W.A., Gunn J.S., Djikeng A., Engidawork E. (2016). Fecal prevalence, serotype distribution and antimicrobial resistance of *Salmonellae* in dairy cattle in central Ethiopia. BMC Microbiol..

[84] Waktole H., Alemie Y., Gizachew M., Yousef A.E., Dessalegn B., Bitew A.B., Alemu A., Gobena W., Christian K., Gelaw B. (2024). Prevalence, Molecular Detection, and Antimicrobial Resistance of *Salmonella* Isolates from Poultry Farms across Central Ethiopia: A Cross-Sectional Study in Urban and Peri-Urban Areas. Microorganisms.

[85] Akenten C.W., Ofori L.A., Mbwana J., Sarpong N., May J., Thye T., Obiri-Danso K., Paintsil E.K., Philipps R.O., Eibach D. (2022). Characterization of Extended-Spectrum Beta-Lactamase-Producing *Escherichia coli* From Domestic Free-Range Poultry in Agogo, Ghana. Res. Sq..

[86] Mekonnen A.S., Gharedaghi Y., Mumed B.A. (2025). Isolation and Antimicrobial Susceptibility Test of Non-typhoidal *Salmonella* from Raw Bovine Milk and Assessments of Hygienic Practices in Gursum District, Eastern Hararghe, Ethiopia. Int. J. Med. Parasitol. Epidemiol. Sci..

[87] Takele S., Woldemichael K., Gashaw M., Tassew H., Yohannes M., Abdissa A. (2018). Prevalence and drug susceptibility pattern of *Salmonella* isolates from apparently healthy slaughter cattle and personnel working at the Jimma municipal abattoir, south-West Ethiopia. Trop. Dis. Travel Med. Vaccines.

[88] Mwambete K.D., Stephen S. (2015). Antimicrobial Resistance Profiles of Bacteria Isolated from Chicken Droppings in Dar es Salaam. Int. J. Pharm. Pharm. Sci.

[89] Cyuzuzo E., Amosun E.A., Byukusenge M., Musanayire V. (2023). Antimicrobial Resistance Profiling of *Escherichia coli* Isolated from Chickens in Northern Province of Rwanda. Afr. J. Biomed. Res..

[90] Tadesse A., Sharew B., Tilahun M., Million Y. (2024). Isolation and antimicrobial susceptibility profile of *Salmonella* species from slaughtered cattle carcasses and abattoir personnel at Dessie, municipality Abattoir, Northeast Ethiopia. BMC Microbiol..

[91] Ndukui J.G., Gikunju J.K., Aboge G.O., Mwaniki J., Kariuki S., Mbaria J.M. (2021). Antimicrobial Resistance Patterns of Selected Enterobacteriaceae Isolated from Commercial Poultry Production Systems in Kiambu County, Kenya. Pharmacol. Pharm..

[92] Mwasinga W., Shawa M., Katemangwe P., Chambaro H., Mpundu P., M’kandawire E., Mumba C., Munyeme M. (2023). Multidrug-Resistant *Escherichia coli* from Raw Cow Milk in Namwala District, Zambia: Public Health Implications. Antibiotics.

[93] Kiiti R.W., Komba E.V., Msoffe P.L., Mshana S.E., Rweyemamu M., Matee M.I.N. (2021). Antimicrobial Resistance Profiles of *Escherichia coli* Isolated from Broiler and Layer Chickens in Arusha and Mwanza, Tanzania. Int. J. Microbiol..

[94] Munyalo J.A., Gebreyes W.A., Mutai W., Anzala O., Ponte A., Merdadus J., Kariuki S. (2020). Detection of Fluoroquinolone and other Multi-drug Resistance Determinants in Multi-drug Resistant Non-Typhoidal *Salmonella* Isolated from Swine. Afr. J. Health Sci..

[95] Haile A.F., Kebede D., Wubshet A.K. (2017). Prevalence and antibiogram of *Escherichia coli* O157 isolated from bovine in Jimma, Ethiopia: Abattoirbased survey. Ethiop. Vet. J..

[96] Kebbeh A., Anderson B., Jallow H.S., Sagnia O., Mendy J., Camara Y., Darboe S., Sambou S.M., Baldeh I., Sanneh B. (2017). Prevalence of Highly Multi-Drug Resistant *Salmonella* Fecal Carriage Among Food Handlers in Lower Basic Schools in The Gambia. Int. J. Nutr. Food Sci..

[97] Ramatla T., Taioe M.O., Thekisoe O.M.M., Syakalima M. (2019). Confirmation of antimicrobial resistance by using resistance genes of isolated *Salmonella* spp. in chicken houses of north west, South Africa. World’s Vet. J..

[98] Kaonga N., Hang’ombe B.M., Lupindu A.M., Hoza A.S. (2021). Detection of CTX-M-Type Extended Spectrum Beta-Lactamase Producing *Salmonella* Typhimurium in Commercial Poultry Farms in Copperbelt Province, Zambia. Ger. J. Vet. Res..

[99] Shilangale R., Kaaya G., Chimwamurombe P. (2016). Antimicrobial Resistance Patterns of *Salmonella* Strains Isolated from Beef in Namibia. Br. Microbiol. Res. J..

[100] Mtonga S., Nyirenda S.S., Mulemba S.S., Ziba M.W., Muuka G.M., Fandamu P. (2021). Epidemiology and antimicrobial resistance of pathogenic *E. coli* in chickens from selected poultry farms in Zambia. J. Zoonotic Dis..

[101] Guesh M. (2017). Prevalence and antimicrobial susceptibility of *Salmonella* species from lactating cows in dairy farm of Bahir Dar Town, Ethiopia. Afr. J. Microbiol. Res..

[102] Beshatu F., Fanta D., Aklilu F., Getachew T., Nebyu M. (2015). Prevalence and antimicrobial susceptibility of *Salmonella* isolates from apparently healthy slaughtered goats at Dire Dawa municipal abattoir, Eastern Ethiopia. J. Microbiol. Antimicrob..

[103] Disassa N., Sibhat B., Mengistu S., Muktar Y., Belina D. (2017). Prevalence and Antimicrobial Susceptibility Pattern of *E. coli* O157:H7 Isolated from Traditionally Marketed Raw Cow Milk in and around Asosa Town, Western Ethiopia. Vet. Med. Int..

[104] WHO (2020). GLASS Whole-Genome Sequencing for Surveillance of Antimicrobial Resistance: Global Antimicrobial Resistance and Use Surveillance System (GLASS).

[105] Sourd G.L., Ricker B. (2025). Cartography for global sustainable development agendas: Informing public policies with geospatial information and knowledge for people, places and planet*. Int. J. Cartogr..

