# Supplementary material for: Antimicrobial Resistance in Selected Foodborne Pathogens in Sub-Saharan Africa: A Systematic Review and Meta-Analysis
Source: Antibiotics (Basel). 2026 Jan 15;15(1):87. doi: 10.3390/antibiotics15010087 (PMC12837378; doi:10.3390/antibiotics15010087)
Supplement: Supplementary file 1 [file antibiotics-15-00087-s001.zip › Supplementary S2b.pdf]

The funnel plot shows significant publication bias as most of the effect sizes are outside the funnel as shows in below (Figure S2(b) ABR prevalence).

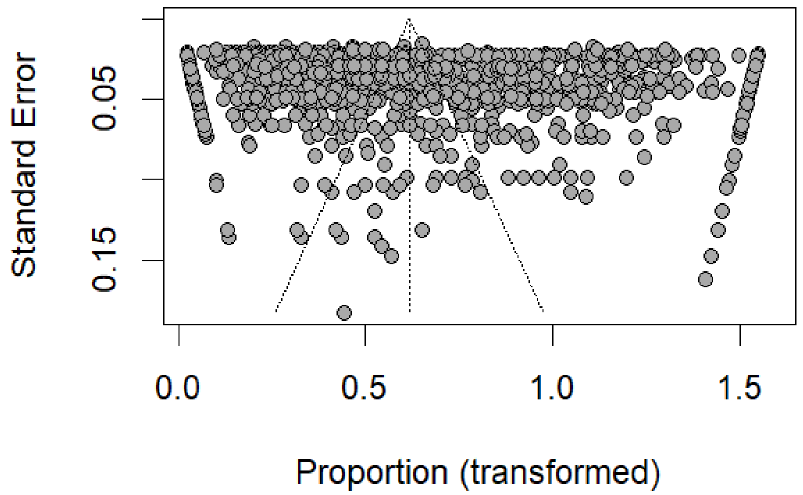

(b) ABR prevalence

Figure S2: Funnel plots results assessing publication bias.
